# Proportion and factors associated with fertility desires among human immunodeficiency virus-positive adults receiving antiretroviral therapy in Northeast Ethiopia

**DOI:** 10.3389/fgwh.2025.1470570

**Published:** 2025-05-19

**Authors:** Abel Endawkie, Biresaw Derese, Kidist Adamu, Getaw Walle

**Affiliations:** ^1^ Department of Epidemiology and Biostatistics, School of Public Health, College of Medicine and Health Sciences, Wollo University, Dessie, Ethiopia; ^2^ Department of Public Health, Zemen Post Graduate College, Dessie, Ethiopia; ^3^ Department of Health System and Management, School of Public Health, College of Medicine and Health Sciences, Wollo University, Dessie, Ethiopia

**Keywords:** proportion, factors associated, fertility desires, human immune deficiency virus positive, adults, antiretroviral therapy, Northeast Ethiopia

## Abstract

**Background:**

The desire to have children can become a significant consideration for many people living with HIV (PLHIV), both men and women, particularly as access to antiretroviral therapy (ART) increases and rates of mother-to-child transmission (MTCT) decline. With the life-prolonging benefits and positive clinical outcomes associated with ART, HIV-positive adults may experience an increased desire to parenting. Nevertheless, research on fertility desires among this demographic remains sparse, especially in Northeast Ethiopia. This study aims to assess the proportion of fertility desires and identify the associated factors among HIV-positive adults receiving care in ART units in Northeast Ethiopia.

**Method:**

A cross-sectional study was conducted from 15 May to 15 June 2022, among 406 individuals living with HIV who were receiving ART in healthcare facilities located in the Meket District of Northeast Ethiopia. The study population consisted of reproductive-age men (18–60 years) and women (15–49 years) who had at least one visit to the ART care units during the study period. Participants were selected through systematic random sampling. Data were collected using self-administered questionnaires. Data entry and analysis were performed using EpiData version 3.1 and Stata version 14.0, respectively. Multivariable logistic regression was employed to identify factors significantly associated with fertility desire, with a *p*-value of <0.05 indicating statistical significance.

**Results:**

The study found that 52.1% of participants expressed a desire for children (95% CI: 47.9, 57.6). Several key factors were associated with fertility desire; specifically, widowed individuals had lower odds of desiring children, while those aged 18–29 had higher odds (AOR: 2.3, 95% CI: 1.1–4.8) compared with those aged 41 and older. Participants aged 30–40 also showed increased odds (AOR: 2.1, 95% CI: 1.3–3.3). Additionally, individuals with one or fewer children had significantly higher odds of fertility desire (AOR: 2.4, 95% CI: 1.2–4.6), and those with 2–3 children had an AOR of 1.9 (95% CI: 1–3.5). A lack of awareness regarding MTCT was linked to an AOR of 2.1 (95% CI: 1–4.4) for expressing a desire for children.

**Conclusion:**

The findings demonstrate a relatively high proportion of fertility desire among HIV-positive adult men and women on ART in Northeast Ethiopia from the national prevalence. This finding underscores efforts that should be directed at individuals aged 18–29 years, who show significantly higher odds of wanting children, along with those aged 30–40 years, those with one or fewer children, and those with two to three children to enhance fertility desire. It is also important to address the needs of widowed individuals, who have lower odds of desiring children, by providing tailored supportive services. The study underscores the necessity for awareness-raising initiatives concerning the prevention of mother-to-child transmission among PLHIV by healthcare providers, highlighting the importance of informed reproductive health choices for PLHIV.

## Introduction

Fertility desire (to have children) may become a viable choice for many people living with human immunodeficiency virus (PLHIV), both men and women, as access to antiretroviral therapy (ART) increases and the risk of mother-to-child transmission (MTCT) declines, Additionally, regaining their health and sexual activity contribute to this desire (1, 2).

The desire and expectation of having children among many people living with HIV has significant implications for the prevention of vertical and heterosexual HIV transmission (3).

Wanting kids is a concern among PLHIV (4) since ART helps extend and improve their quality of life (4, 5).

The future aspirations of HIV-positive individuals to have children greatly affect the risk of HIV transmission to their partners and unborn children (6). Improved access to antiretroviral therapy (ART) is likely to increase the desire among this population (7).

In relation to the fertility desire of PLHIV, a study conducted in different parts of Nigeria found that approximately 65.8% of participants expressed a desire to have more children (8). Other studies reported that individuals who experienced improved health while on ART were significantly associated with the desire to have children. In a cohort study at AIDS care centers, 89% of participants who were on antiretroviral therapy had fertility desire (9).

According to studies in different parts of Ethiopia, HIV/AIDS is most prevalent among women aged 20–29 years and men aged 20–39 years (10, 11). A study conducted in Nigeria, South Africa, Zimbabwe, and Ethiopia on HIV-positive women found that their desires to have children (12, 13) varied.

Research in Ethiopia suggests that the proportion of individuals living with HIV who express a desire to have children ranges from 30% to 70% (14, 15). This desire can be influenced by factors such as age, marital status, ART, prevention of mother-to-child transmission (PMTCT), and cultural beliefs (14–16). However, the evidence regarding the fertility desires of individuals living with HIV in Ethiopia is limited, particularly in the northeastern region of Amhara, Ethiopia. Research indicates that while many people living with HIV (PLHIV) express a desire to have children, the extent of this desire and the factors influencing it are not well understood. Factors such as ART service, PMTCT awareness, and individual and demographic factors may play significant roles in shaping these desires, but comprehensive studies specifically addressing these aspects among PLHIV on antiretroviral therapy (ART) in Northeast Ethiopia are sparse. Therefore, this study aims to assess the proportion of fertility desire and associated factors among PLHIV following ART care units in ART clinics in Northeast Ethiopia.

## Methods

### Study design, area, and period

An institutional-based cross-sectional study was conducted in Meket District Northeast Ethiopia from 15 May to 15 June 2022. Meket District, located in Northeast Ethiopia's Amhara region, provides antiretroviral therapy (ART) services to people living with HIV through its two health facilities equipped to deliver comprehensive care, including counseling, testing, and ongoing ART treatment. These facilities also provide services to patients from the neighboring Tigray region. With a significant patient population relying on these services, local health authorities actively engage the community to raise awareness about HIV and encourage individuals to seek testing and treatment, thereby reducing stigma.

### Source and study population

The source population included all PLHIV receiving care at the ART unit and those who had taken at least one visit to the ART unit during the study period. The study population consisted of PLHIV within the reproductive age—women aged 15–49 years and men aged 18–60 years.

### Sample size determination and sampling technique

The sample size was determined using a single population proportion formula by taking the proportion of HIV-positive individuals who received ART treatment and had the desire to have children 40% (from a study conducted in Amhara region referral hospitals in 2017) (17). A 5% margin of error, 95% confidence interval, and 10% non-response rate were assumed. *n* = (*z*·*α*/2)^2^·*p*·(1 − *p*)/*d*^2^. The final sample size for the study was 406. Two health facilities with ART clinics in Meket District, Northeast Amhara, Ethiopia, provided ART services to a total of 1,162 patients. These samples were proportionally allocated to each of the two facilities. Using the medical record number (MRN) registration of clients, a systematic random sampling technique was employed, selecting participants at every *k*-th interval after a random start. The value of *k* was determined by dividing the total number of PLHIV by the sample size (1,162/406 = 3), resulting in the selection of every third participant during the data collection month.

### Variable measurement and definitions

The dependent variable fertility desire is categorized and coded as 1 = “yes” and 0 = “no”.

The independent variables included sociodemographic characteristics (such as age, sex, income, marital/relationship status, education level, religion, occupation, and ethnicity) and factors such as the number of living children, partner's HIV status, partner’s desire for children, and the duration since HIV diagnosis.

**Fertility desire:** It is a psychological state in which someone has the personal motivation to have a child. In this study, participants who have the motivation to have more children in the future were considered to have a fertility desire, while those who do not have the motivation to have more children were considered not to have fertility desire during this period (18).

**PLHIV on ART follow-up care**: All PLHIV who have at least one visit to the ARV treatment units for care and were receiving ARV treatment (19).

**HIV-positive men**: Men living with HIV aged between 18 and 60 years taken from ART care registration at the study institutions (19).

**HIV-positive women**: Women living with HIV aged between 15 and 49 years take from ART care registration at the study institutions (19).

### Data collection techniques and procedures

Data were collected using self-administered questionnaires. The questionnaire was adapted from different literature (17, 20–23) in English and then translated into Amharic language and then back to the English language for analysis. Face-to-face interviews were used to collect characteristics such as sociodemographic characteristics, child desire information, and ART treatment conditions to prevent recall bias. A questionnaire was administered by healthcare providers working in the ART care units after getting a 1-day training on how to collect the data. Two supervisors were also recruited and closely followed the data collection process with the principal investigator.

### Data quality control measures

To ensure the quality of data, data collectors and supervisors were trained for 1 day. During the data collection procedures, all the collected data were reviewed and checked daily for completeness. The questionnaire was pretested in the ARV treatment units selected for the study (at Kombolcha Hospital) which was out of the selected ART sites. The pretest was conducted on approximately 10% of the total sample size, and the validity and reliability of the instrument were checked. After the pretest had been conducted, corrections were made to the missed variables and ambiguous questions. In the data collection process, the privacy and confidentiality of the respondents were maintained by excluding their names from the interview, and after collecting the questionnaire from the study sites, manual editing and coding were undertaken to enhance data quality.

### Data processing, analysis, and interpretation

The data were checked for completeness and consistency in the field, coded and entered into EpiData version 3.1 software, and exported to STATA version 17 software for processing and analysis. Consistency and completeness of data were checked using frequency and two-by-two tables. Descriptive statistics such as frequency distributions and measures of central tendencies were calculated for dependent and independent variables. Variables with a *p*-value of <0.25 in bivariable logistic regression were candidates for multivariable analysis and transferred to multivariable analysis. Model fitness was checked using the Hosmer and Lemeshow goodness of fit test, and multicollinearity was checked using the variance inflation factor (VIF). There is no multicollinearity with an average VIF of 3.4. Finally, variables with a *p*-value of <0.05 in the multivariable analysis of binary logistic regression were taken as factors significantly associated with fertility desire.

### Ethical considerations

Ethical approval was obtained from the Institutional Review Board of Zemen Postgraduate College Public Health Department. An official letter of cooperation was given to the health administrative offices of Meket District of the health facility ART clinic. Permission letter was acquired from the two administrative health offices. Each study participant gave written informed consent to confirm their willingness to participate in the research after being told of the study's goal and purpose. Written informed consent was presumably obtained from legally authorized representatives, such as parents and/or legal guardians, for respondents who were incapable of providing written informed consent, such as illiterate men and women. In addition, participants were made aware that their participation in the study was entirely voluntary and that they might leave at any moment if the questionnaire did not feel comfortable. Participants’ names were not entered into the questionnaire, and the data were password-protected and stored on a computer to maintain privacy and secrecy. During the data collecting period, the study subjects received critical advice regarding ART care and diet from the data collectors, and all methods and data were performed based on Helsinki principles and Ethiopian research guidelines.

## Results

### Sociodemographic characteristics of the respondents

In this study, approximately 401 respondents participated making a response rate of 98.7%. Of the 401 respondents, 232 (57.9%) were females. The age of respondents ranges from 25 to 56 years (mean and SD, 39.6 ± 7.885, respectively). Approximately 181 (45.1%) of participants were aged greater than 41 years, approximately 259 (64.6%) were rural residents, 165 (41.1%) had primary and above educational status, 386 (96.3%) belonged to the Orthodox Christian religion, and 261 (65.1%) were married (Table 1).

**Table 1:** Sociodemographic characteristics of PLHIV attending ARV treatment units, in Meket Districts, North Wollo Zone Amhara region 2022.

Variable	Category	Frequency	Percent
Age	18–29	52	13
	30–40	168	41.9
	≥41	181	45.1
Sex	Male	169	42.1
	Female	232	57.9
Residence	Urban	142	35.4
	Rural	259	64.6
Educational status	Unable to read and write	131	32.7
	Can read and write	105	26.2
	Primary and above education	165	41.1
Marital status	Married	261	65.1
	Single	51	12.7
	Widowed	46	11.5
	Divorce	43	10.7
Monthly income	≤5,000	242	60.3
	>5,000	159	39.7
Occupational statues	Housewife	94	23.4
	daily labor	88	21.9
	Merchant	50	12.5
	Government	19	4.7
	Farmer	91	22.7
	Private employ	59	14.7
Religion	Orthodox	386	96.3
	Muslim	15	3.7

### Sexual behavior and condom use of PLHIV on ART treatment

A total of 290 study participants (74.2) were sexually active within the last 6 months, of whom 131 (45.2%) used condoms (Table 2).

**Table 2:** Sexual behavior and condom use of PLHIV attending ARV treatment unit Meket District ART clinic Northeast Ethiopia 2022.

Characteristics	Category	Frequency	Percent
Sexually active in the past 6 months	Yes	290	74.2
	No	101	25.8
Use a condom (*N* = 290)	Yes	131	45.2
	No	159	54.8
How often to use a condom (*N* = 124)	Always	55	42
	Sometimes	76	58

### HIV-related characteristics of PLHIV attending ART care

The study indicated that 351 (87.5%) had awareness of PMTCT, and approximately 167 (47.6%) of the study participants reported that HIV can be transmitted from mother to child during pregnancy (Table 3).

**Table 3:** HIV-related characteristics of PLHIV attending ARV care in Meket District ART clinic Northeast Ethiopia 2022.

Variables	Category	Frequency	Percent
HIV transmitted from mother to child	Yes	351	87.5
	No	50	12.5
Time of HIV transmission	During pregnancy	167	47.6
	During delivery	129	36.8
	During breastfeeding	55	15.6

### Proportion of fertility desire among PLHIV

The proportion of fertility desire among PLHIV in Meket District ART clinic Northeast Ethiopia was 52.1% (95% CI: 47%–57.6%).

### Factors associated with fertility desire among adult patients livid with HIV/AIDS

In bivariable logistic regression analysis residence, educational status, age, marital status, number of children, awareness of MTCT, and discussion with healthcare providers related to reproductive and sexual health at a *p*-value of <0.25 were candidates for multivariable binary logistic regression.

After controlling other factors constantly using multivariable analysis, participants in the age group of 18–29 years, being widowed, having ≤1 child, and lacking awareness of mother-to-child transmission were found to be significantly associated factors with fertility desire at *p* < 0.05. Individuals who were in the age group 18–29 and 30–40 years had 2.34 times (AOR = 2.34, 95% CI: 1.139–4.794) and 2.10 times (AOR = 2.102, 95% CI: 1.321–3.344) higher odds of desiring fertility, respectively, as compared with those who were aged 41 years and above. The odds of having fertility desire among PLHIV who had ≤1 child and 2–3 children were 2.43 times (AOR = 2.426 95% CI: 1.27–4.63) and 1.94 times (AOR = 1.94, 95% CI: 1.05–3.59) higher than PLHIV who had >4 children. Fertility desire among widowed individuals was 65.4% (AOR = 0.35, 95% CI: 0.15–0.78) less likely than married ones. Study participants who were not aware of mother-to-child transmission 2.18 times (AOR = 2.18, 95% CI: 1.08–4.4) had higher odds of desiring children as compared with those who had good awareness about mother-to-child HIV transmission (Table 4).

**Table 4:** Multivariable logistic regression analysis for associated factors of fertility desire PLHIV in Meket District ART clinic Northeast Ethiopia.

Category		Fertility desire		COR	AOR	*p*-value
		Yes	No			
Marital status	Married	142	119	Ref	Ref	Ref
	Single	35	16	1.83 (0.97–3.48)	1.60 (0.77–3.32)	0.20
	Widowed	12	34	0.29 (0.15–0.60)	0.35 (0.15–0.78)	0.011*
	Divorce	20	23	0.73 (0.38–1.39)	0.97 (0.48–1.97)	0.94
Age	18–29	34	16	3.35 (1.67–3.94)	2.33 (1.14–4.79)	0.021&
	30–40	104	64	2.56 (1.71–4.01)	2.10 (1.32–3.34)	0.002*
	≥41	71	112	Ref	Ref	Ref
Number of children	≤1	88	56	3.20 (1.77–5.80)	2.426 (1,271–4.631)	0.007*
	2–3	97	87	2.28 (1.29–4.02)	1.94 (1.05–3.59)	0.035*
	≥4	24	49	Ref	Ref	Ref
Awareness on PMTCT^a^	YES	175	176	Ref	Ref	Ref
	NO	34	16	2.14 (1.14–4.01)	2.18 (1.08–4.41)	0.03*

AOR, adjusted odd ratio; COR, crude odd ratio; Ref, reference.

^a^ PMTCT, prevention of mother-to-child transmission.

* Significant at *p* < 0.05.

## Discussion

This study assessed the prevalence of fertility desire and the factors associated with it among people living with HIV (PLHIV) in Northeast Ethiopia.

The findings indicate that the prevalence of fertility desire among PLHIV in this region is 52.1% (95% CI: 47.9–57.6). This result aligns with a study conducted at Arba Minch Hospital, which reported that 53.5% of participants expressed a desire for fertility (14). The similarity might be due to the similar study design and communities' relatively similar behavior, culture, and socioeconomic status. In this study, the prevalence of fertility desire was higher than in Amhara region referral hospitals (40.8%) (17) and the national prevalence of fertility desire of PLHIV in Ethiopia (15). The difference may stem from several factors, including the sociodemographic characteristics of the participants, such as age, education, income, and marital status, which can influence fertility perceptions. Inadequate communication with healthcare providers during data collection may also play a role, as poor exchanges can lead to misunderstandings about fertility options. Additionally, variations in sample size between studies can affect the reliability of the findings, with larger samples typically offering more accurate representations.

In this study, participants aged 18–29 and 30–40 years were found to be 2.33 and 2.1 times more likely to express a desire for fertility, respectively, when compared with individuals aged 41 and older. This finding is consistent with various studies conducted in the Oromia region, specifically in the Horro Guduru Zone, which supports these observations (23). One possible explanation for this trend is that older respondents may have already reached or are nearer to achieving their desired family size compared with their younger counterparts.

In this finding, participants who had less number of children had higher fertility desires than those who had more children. This was supported by studies done in Ethiopia, Nigeria, and Brazil (13, 14, 23, 24). The reason behind this finding may be that participants with fewer children often have a greater desire to expand their families and fulfill their fertility goals. In contrast, those with more children might feel that they have already achieved their desired family size and therefore have less impetus to seek additional children. This finding implies that healthcare providers and policymakers should consider the fertility desires of individuals with HIV when designing reproductive health programs and services. Additionally, addressing the specific needs and desires of these individuals may contribute to more effective family planning strategies and ultimately improve reproductive health outcomes in the community.

In this study, we found that widowed participants have less odds of having fertility desire than married individuals. This result was supported by a study conducted in Nekemt East Welega Zone (25). The possible explanation might be a single individual free from peer pressure and the absence of shared responsibility for carrying a child.

In this study, awareness of PMTCT emerged as a significant factor influencing fertility desire. Participants who were unaware of mother-to-child transmission (MTCT) were 2.18 times more likely to express a desire for fertility compared with those who were informed about MTCT. This finding aligns with research conducted in other regions of Ethiopia (15, 22, 26). The possible explanation might be individuals who did not understand the risk of HIV transmission from mother to child increasing fertility desire. This study found that those lacking discussion with health care providers about reproductive health were 3.3 times more likely to have fertility desires as compared with those who discussed with health care providers. This finding is supported by the previous study findings (20, 21). This finding implies that open communication with healthcare providers can significantly influence individuals' understanding of reproductive health. When patients engage in discussions about their reproductive options, they are more likely to receive tailored information and guidance, which can help shape their fertility desires.

Study limitation: The limitation of this study lies in its cross-sectional design, which hampers our ability to establish causality. Additionally, the design is susceptible to recall bias and social desirability bias, as data were collected by healthcare providers in ART clinics to ensure the confidentiality of the participants.

## Conclusions

The findings demonstrate a relatively high proportion of fertility desire among HIV-positive adult men and women on ART in Northeast Ethiopia from the national prevalence. This finding underscores that efforts should be directed at individuals aged 18–29 years, who show significantly higher odds of wanting children, along with those aged 30–40 years, with one or fewer children and those with two to three children to enhance fertility desire. It is also important to address the needs of widowed individuals, who have lower odds of desiring children, by providing tailored support services. The study underscores the necessity for awareness-raising initiatives concerning the prevention of mother-to-child transmission among stakeholders and healthcare providers, highlighting the importance of informed reproductive health choices for individuals living with HIV.

## Data Availability

The original contributions presented in the study are included in the article/Supplementary Material, further inquiries can be directed to the corresponding author.
